# What do we mean when we say an operation works?

**DOI:** 10.1093/jhps/hnw019

**Published:** 2016-06-10

**Authors:** Richard Villar

At a conference the other day, while hotly debating the role of hip arthroscopic surgery in the management of osteoarthritis, a friend and colleague announced to the assembled throng, ‘We know that hip arthroscopy doesn’t work’. You would not have expected the Editor-in-Chief of this journal to remain silent at that moment and I certainly was not. However, what the statement did highlight, of course, is what do any of us mean when we say an operation works?

The first thing to remember is that hip arthroscopy is not an operation, it is a technique. One major issue, to my mind, is the apparent obsession that the success, or otherwise, of hip arthroscopy is intimately linked to femoroacetabular impingement (FAI). That close association has brought with it many problems. I hear of regions, indeed countries that are considering rejecting hip arthroscopic surgery based solely on the assessment that it may not work for FAI. Believe me when I say, if FAI disappeared tomorrow there would still be plenty enough for a hip arthroscopic surgeon to do. There are some surgeons around who were arthroscoping hips well before FAI was ever reinvented. How about stabilization, labral repair, labral grafting, chondral repair, loose body removal, synovectomy, sepsis, debridement, extra-articular techniques and plenty more besides? For the future of hip arthroscopy, certainly when seen from the viewpoint of this ageing orthopaedic surgeon, the sooner we can unlink hip arthroscopy from FAI in the managerial mind, so much the better. By all means lay out an algorithm for the management of FAI but do that for FAI not for hip arthroscopy.

But to say an operation works depends on who you are. One surgeon may feel that the definition of success is if a patient makes it to the Recovery Area after the procedure while another is seeking 2 years of pain relief to justify recommending surgery. Meanwhile the hospital manager might prefer a short stay, no chance of readmission, and as little expenditure as may seem reasonable. The patient, who is after all the major player in all of this, might have an entirely different view. Only the other day a 40-year-old patient came to my clinic with cam FAI. I offered him hip arthroscopic surgery based on an 80% chance of success. To me that seemed reasonable and reflected my own performance figures, not those borrowed from elsewhere. However, I also said to the patient that he should write down what he sought, on the assumption everything went perfectly. Word for word, his expectations were as follows:

‘After my perfect operation I would not require a hip replacement in later life. My mobility would improve such that I could touch my toes. And I would continue (or restart) my running or even play the odd game of football. I would not get hip pain in bed or getting out of the car. I would not feel like a sixty-year-old’.

Oh dear. My problem, however many of these I undertake, is that I cannot promise him any of this, much as I would wish differently. So we have a patient/surgeon disconnect, created simply because we see things from different points of view. Hip arthroscopy is not unique in this dilemma. For total knee arthroplasty it has been shown that patients have expectation scores higher than those of their surgeon [1]. We surgeons tend also to overestimate the progress of recovery from injury [2] and underestimate the significance of any complications [3] when compared with our patients. Three factors, certainly in respect of joint replacement, broadly determine a patient’s overall satisfaction after surgery and these are meeting preoperative expectations, achieving satisfactory pain relief, and a satisfactory hospital experience [4]. I am not persuaded that any of the many scores we each use fully reflects such things. Ultimately the simplest way of assessing postoperative outcome might be to ask, ‘Are you happy with your hip—yes or no?’

The other concern I have is what do we regard as suitable evidence that an operation works? If we find, say, ten Level 4 studies each reaching an identical conclusion but one Level 1 study saying something completely different, what do we believe? I came across a paper the other day that said there was fair evidence to support the use of hip arthroscopy for the treatment of FAI but that other indications for the technique lacked adequate evidence-based support [5]. The observation was, I suppose, reasonable but the paper itself was only Level 4. So we have a Level 4 systematic review saying that other Level 4 studies are inadequate. Somehow that does not compute. Meanwhile I take much heart from the paper in this very journal, *JHPS*, declaring that at the annual ISHA meetings the levels of evidence for the articles and posters presented worked out as Level 1: 10.1%, Level 2: 12.8%, Level 3: 30.1% and Level 4: 47.0%. Slowly the number of Level 1 and 2 articles was increasing in tandem with a similar decline in the number of Levels 3 and 4 [6]. This is excellent to see.

Turning to the last issue of *JHPS*, there were again plenty of tremendous papers to read as our journal steadily becomes *the* place to see your work published. The other day one of the household names in the subspecialty turned to me to say that *JHPS* was the only journal that he read from cover to cover. For all others he just dipped in and out but, to him, *JHPS* was where it was at. Thanks so much to all of you who have helped this take shape and who appear to clearly agree with him. But in our last issue I was again glued to the papers on orthobiologics as I declare a vested interest and, anyway, orthobiologics is an area that is slowly taking greater hold. There is the review article by Al Stubbs *et al*. [7] and the other by Rodrigo Mardones and Catalina Larrain [8]. Both are fantastic pieces and I commend them to you, as I do for all the other articles in that issue of *JHPS*.

And in this issue, our latest, what have we got? Again, there is so much to offer it is difficult to know where to start. However, I confess to a degree of voyeurism, if that is the right word, when I read Thomas Byrd’s report on a cohort of orthopaedic surgeons who underwent hip arthroscopic surgery. We are a sickly lot by the look of things and do need to modulate our expectations [9]. Then do also have a good read of the paper on microfracture by Green et al. Basically, could we have it all wrong? I wager you will take sides once you have read what they say [10].

So once again welcome to this, the latest issue of *JHPS*, a journal that would be impossible without every last one of you playing his or her part. It goes without saying that I/we/everyone involved is enormously grateful to you. Enjoy every word and be sure to read us from cover to cover, or whatever the digital equivalent might be.

My very best wishes to you all.
